# Health and economic impact of hepatitis B vaccination in the national immunization program in Belarus, Georgia, Kyrgyzstan, Republic of Moldova, Turkmenistan and Uzbekistan—a modelling study

**DOI:** 10.3389/fpubh.2025.1567033

**Published:** 2025-10-06

**Authors:** Maryam Sadeghimehr, Liudmila Mosina, So Yoon Sim, Siddhartha Sankar Datta

**Affiliations:** ^1^WHO Regional Office for Europe, Copenhagen, Denmark; ^2^WHO Regional Office for Europe, Copenhagen, Denmark; ^3^Department of Immunization, Vaccines and Biologicals, WHO Headquarters, Geneva, Switzerland; ^4^WHO Regional Office for Europe, Copenhagen, Denmark

**Keywords:** hepatitis B virus, vaccination strategies, chronic liver disease, impact analysis, mathematical modelling, preventive strategies

## Abstract

Hepatitis B (HBV) remains a major public health concern in the WHO European Region, causing approximately 32,000 deaths annually as of 2022. Fortunately, HBV vaccination is highly effective in preventing infection. The WHO Regional Office for Europe established criteria for countries to be validated in reaching interim targets for HBV control through immunization, advancing toward the goal of hepatitis elimination. Belarus, Georgia, Kyrgyzstan, Moldova, Turkmenistan, and Uzbekistan conducted national HBV serosurveys, which revealed a significant reduction in chronic HBV prevalence among individuals born after the introduction of universal HBV vaccination in their national immunization programs. We analyzed these serosurvey data and developed a mathematical model to estimate the impact of various vaccination scenarios on HBV-related deaths and severe liver disease. The vaccination scenarios included the current vaccination strategies, as well as two other full-vaccination scenarios—one beginning at vaccine introduction and another starting in 2021 and also a no vaccination scenario. The vaccination scenarios reflect the programmatic context in the countries such as administration of hepatitis B immunoglobulin to infants born to HBsAg positive mothers and hepatitis B sero-prevalence among vaccinated cohorts of children. In the optimal scenarios, we assumed 95% coverage for all these interventions. We used two types of analysis: a birth cohort analysis tracking each cohort until death and a calendar-based analysis assessing the HBV burden annually from the year of vaccine introduction in each country up to 2040. Additionally, we estimated cost savings by calculating the avoided treatment costs and the costs associated with time spent in different health states, based on the outcomes of the calendar-based analysis. Our findings suggest that the existing vaccination programs have reduced HBV-related mortality in birth cohorts by 96.9 to 98.85%. The calendar-based analysis showed that vaccination programs have averted 24.08% (95% CI: 21.73–26.43%) of HBV-related deaths since their introduction, with the confidence interval reflecting variation in outcomes across the countries. Furthermore, high vaccination coverage resulted in a saving between US$ 6.07 million and US$ 34.8 million in treatment costs in the countries. This study underscores the importance of timely vaccination strategies as a powerful preventive measure in combating HBV globally.

## 1 Introduction

Viral hepatitis B poses a significant public health threat in the WHO European Region (Region). It is estimated that in 2022, hepatitis B was responsible for 32,000 deaths and has resulted in 18,000 new infections (1). To urgently expand access to life-saving interventions and prevent new infections, the Region adopted the Regional action plans for ending AIDS and the epidemics of viral hepatitis and sexually transmitted infections 2022–2030 (2) during the 72nd session of the WHO Regional Committee for Europe. The adoption of the action plan led to the setting of the goal to end viral hepatitis as a major public health problem in the Region which included universal access to hepatitis B vaccines and improved services for testing pregnant women to prevent perinatal transmission of hepatitis B virus (HBV).

As of 2022, 50 of the 53 Member States (86%) in the Region successfully implemented universal hepatitis B immunization programmes (3). The WHO Regional Office for Europe (Regional Office) provides continuing support to Member States in implementing the action plan and monitors progress.

towards reaching its goals. The European Technical Advisory Group of Experts on Immunization advises the Regional Office on the operational aspects of strengthening hepatitis B control and, through its Working Group on Hepatitis B, validates achievement of the interim targets for hepatitis B control through immunization, thereby progressing towards the path to hepatitis elimination.

By 2023, nine countries in the Region were validated as having reached the hepatitis B control targets. Among them, Belarus, Georgia, Kyrgyzstan, the Republic of Moldova, Turkmenistan, and Uzbekistan conducted nation-wide representative hepatitis B serosurveys. The findings from these serosurveys demonstrated a substantial reduction in the prevalence of chronic hepatitis B among individuals born in these countries after the introduction of universal hepatitis B vaccination in their national immunization schedule (4).

To demonstrate the value of investing in childhood hepatitis B vaccination by the Ministries of Health, the Regional Office conducted a modeling study to transpose the data from the hepatitis B seroprevalence studies into measurable health and financial benefits for the national health systems. This model evaluated the impact of hepatitis B vaccination, demonstrated through low levels of HBsAg seroprevalence, by computing the number of hepatitis B related deaths averted and savings from the treatment of hepatitis B related complications in these countries.

Several mathematical models have been developed and used to measure hepatitis B progression and transmission (5–16). In 2019, the Global Burden of disease (GBD) Hepatitis B Collaborators (7), conducted a comprehensive estimation of global, regional, and national prevalence of hepatitis B virus (HBV), including mortality and disability-adjusted life-years due to HBV. In 2016, Edmunds et al. (5) using a static model assessed the impact of hepatitis B vaccination on severe HBV-related disease, specifically liver cancer and cirrhosis. Their detailed estimates were based on the data from China, Gambia and South Korea (5, 6). Using a Markov model, Lu et al. (13) estimated the long-term cost-effectiveness of universal newborn hepatitis B vaccination in China, a country with high endemicity of hepatitis B. Chen et al. (8) provided a comprehensive economic evaluation of infant HBV vaccination combined with Hepatitis B immunoglobulin (HBIG) in China. In 2016, Razavi et al. (16) used a dynamic HBV transmission and progression model to estimate the prevalence of HBV for the countries and the region including the impact of prophylaxis and treatment on disease burden. In 2005, Goldstein et al. (11) developed a model to estimate the impact of global HBV vaccination in reducing HBV-related morbidity and mortality.

The mathematical models linked to hepatitis B (5–16) mostly focus on the disease burden within a country or the focus is related to economic evaluations of hepatitis B vaccination without estimating the impact of vaccination. An estimation of the impact of vaccination on the hepatitis B disease burden is not available in any of the six countries in the Region which have conducted the seroprevalence studies. Using the findings from hepatitis B serosurveys in the six countries in the Region, we developed a model to estimate the number of hepatitis B cases and deaths that have been averted until date in these countries and those which will be further averted by sustaining high hepatitis B vaccination.

## 2 Materials and methods

### 2.1 Model structure and inputs

We developed a mathematical model using a Markov chain with a one-year cycle to simulate the life course of individuals from birth to death. The model assumes that at any given time, susceptible individuals may progress to the acute phase and subsequently transition to the chronic phase, depending on the prevailing force of infection within the population.

**Figure 1:**
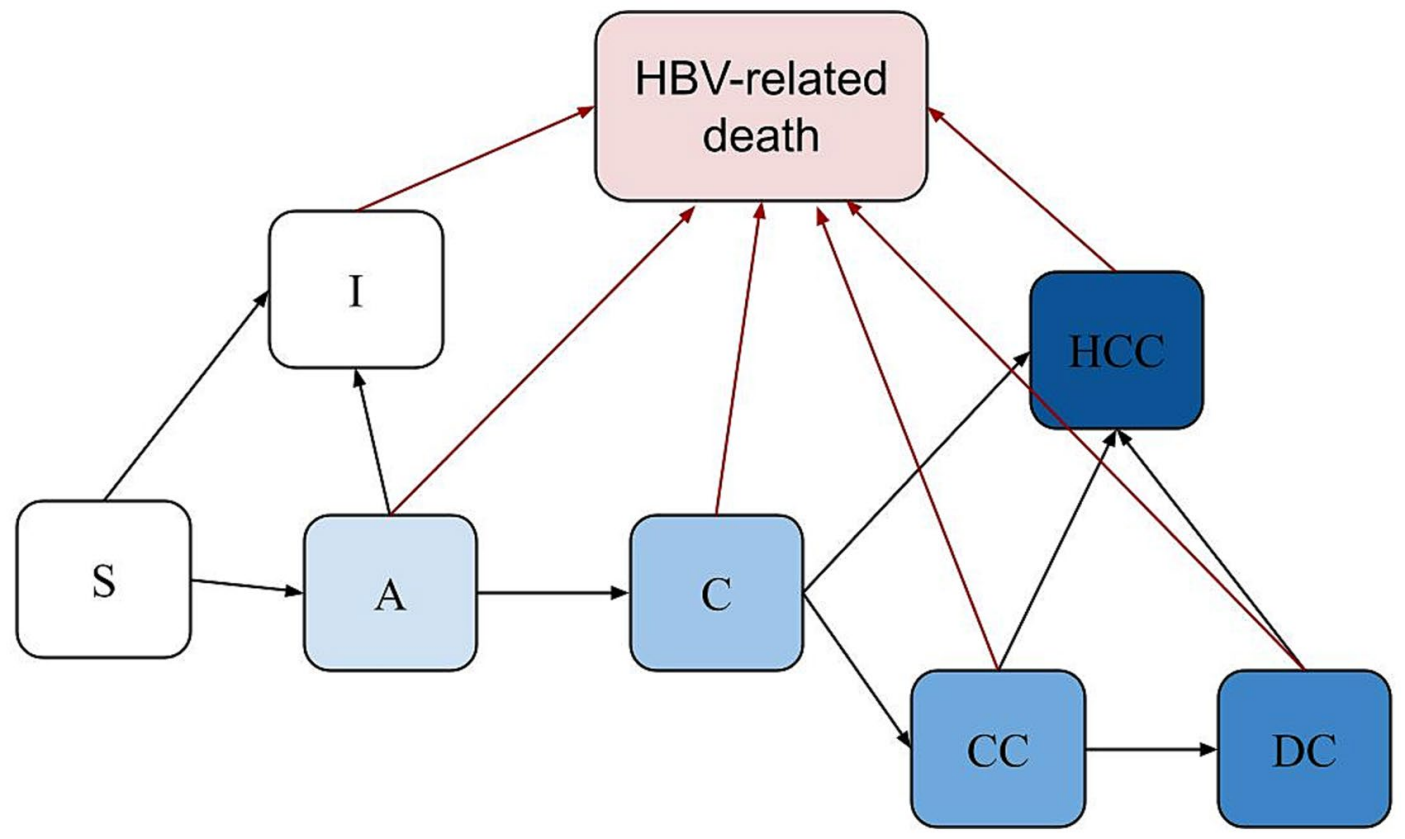
Structure of the model. S, Susceptible; A, Acute; I, Immune; C; Chronic; CC, Cirrhosis; DC, Decompensated Cirrhosis; HCC, Hepatocellular carcinoma; Death from causes other than HBV can occur in any health state, based on country-specific life tables (not displayed in the figure for simplicity). The newborns were categorized into two groups based on their mothers’ HBV infection status.

We considered two sources of HBV infection incidence: (1) mother-to-child transmission at birth, which is based on seroprevalence data from the countries under study and is incorporated according to maternal HBV status and antiviral treatment, with separate probabilities for newborns of untreated and treated HBV-positive mothers, as shown in Table 1; and (2) horizontal transmission through exposure to the virus later in life, with incidence rates calibrated by age and HBV prevalence for each year and country. For each country, age-specific incidence rates were iteratively adjusted and weighted to match observed prevalence across age groups, using least-squares minimization. In both pathways, the risk of infection is reduced by the efficacy of the HBV vaccine, depending on vaccination coverage, efficacy and timing. Infected individuals may develop severe liver disease over their lifetimes, with severity represented by cirrhosis, decompensated cirrhosis (DC), and hepatocellular carcinoma (HCC). Death in the model can occur due to hepatitis B-related causes or other factors. Annual country-specific background mortality rates, obtained from the Global Health Observatory data repository, were applied to age groups across populations. Additionally, specific mortality rates were applied to populations with cirrhosis, DC, and HCC. Figure 1 provides a visual representation of the model’s structure.

**Table 1:** Input parameters.

Parameter	Value	Source
Disease parameters		
Progression rate from cirrhosis to death	0.039 Piecewise cubic polynomial functions used to model age-dependent rates for Uzbekistan^*^	(8, 11, 18)
Progression rate from DC to death	0.39	(8, 17, 18)
Progression rate from HCC to death	0.56 Piecewise cubic polynomial functions used to model age and gender dependent rates for Uzbekistan^**^	(8, 11, 17, 18)
Probability of death due to fulminant Hepatitis	67% in 0.5% of infected cases at birth	(37)
Rate of developing chronic infection:	80–90%	(38)
Infants infected during the first year of life	30–50%	
Children infected between the ages of 1 and 5 years	95%	
Progression rate from chronic hepatitis to cirrhosis	0.005	(13, 16)
Progression rate from chronic hepatitis to HCC	0.0006	(16)
Progression rate from cirrhosis to DC	0.054	(16)
Progression rate from cirrhosis to HCC	0.024	(16)
Progression rate from DC to HCC	0.024	(16)
Vaccine efficacy	95%	(39, 40)
Vaccine efficacy among newborns with HBV + treated mothers	95%	(39, 40)
Vaccine efficacy among newborns with HBIG treatment	97%	(39, 40)
Risk of infection among newborns with HBV + mothers:		
who did not use antiviral treatment	0.875	(8)
who used antiviral treatment	0.253	(8, 41)
Risk of infection among newborns with HBV-mothers:		
for the years before the introduction of HBV vaccine	0.05	(6, 8, 42)
for the years after the introduction of HBV vaccine	0.001	(43), Assumption
Treatment response rate	95%	(44–46)
Country-specific parameters		
Vaccine coverage	hepatitis B vaccination coverage of each of the countries in reference	WHO/UNICEF estimates of national immunization coverage
Catch-up vaccination coverage scenario included in model for each country	Belarus: Catch-up vaccination of 13-year-old children between 1999 and 2015. Kyrgyzstan: Catch-up vaccination of adults aged 23–65 years. A catch-up vaccination campaign was conducted in 2022 to address the drop in vaccine coverage during the COVID-19 pandemic. Georgia: Catch-up vaccination of 12-year-olds in 2000 and for adolescents between 2009 and 2012. Moldova: Catch-up vaccination among the 1988–1992 birth cohorts in 2004 and 2005. Turkmenistan: No catch-up vaccination conducted Uzbekistan: No Catch-up vaccination conducted	(12, 16, 20, 21)
Treatment rate	Country-specific values as of 2016	(16)
Diagnosis rate	Country-specific values as of 2016	(16)
HBIG treatment rate	Georgia: 43% in 2016, 82% in 2019 and 87% in 2020 Belarus: 48% Others: 0	(12, 16, 20, 21)
Antiviral treatment rate	Belarus: Yes Others: No	(12, 16, 20, 21)
Total population by age (per year)	World population prospects	(22)
Total population at birth (per year)	World population prospects	(22)
Background mortality data	Country-specific background mortality data were obtained from the Global Health Observatory data repository (WHO)	(19)

We projected the newborn data to obtain population estimates for the future.

* For individuals aged less than 38: $0.015*(\mathrm{age}-4)-0.069{\left(\mathrm{age}-4\right)}^{2}+0.0028{\left(\mathrm{age}-4\right)}^{3}$ and for individuals aged 38 or older: $-14.436*(\mathrm{age}-4)-0.6483{\left(\mathrm{age}-4\right)}^{2}+0.004756{\left(\mathrm{age}-4\right)}^{3}.$

** For females: $0.099383*(\mathrm{age}-4)-0.004356{\left(\mathrm{age}-4\right)}^{2}+0.003179{\left(\mathrm{age}-4\right)}^{3},$ for males: $3.316492*(\mathrm{age}-4)-0.204495{\left(\mathrm{age}-4\right)}^{2}+0.003615{\left(\mathrm{age}-4\right)}^{3}.$

The model used the following population-specific data to calculate disease burden and the reduction in disease burden from hepatitis B vaccination: annual size of birth cohorts, total population of the country, sex ratio at birth, prevalence of HBV infection among pregnant women, prevalence of HBV in the general population, annual hepatitis B vaccination coverage, rate of hepatitis B diagnosis, treatment rate and overall background mortality rate of the population (as a competing risk factor). To parameterize and populate the model, we conducted a literature review to gather country-specific and regional/global demographic and epidemiological data (7, 8, 11, 16–18). Additionally, the required epidemiological data and the hepatitis B vaccination coverage were collated from various data sources as the WHO Global Health Observatory data repository (19), the Fourth & Fifth Meeting reports of the European Technical Advisory Group of Experts Working Group on hepatitis B (12, 20), and meeting reports from the coalition for global health elimination (21) and, population data from the United Nations, World Population Prospects 2022 (22). Table 1 outlines the input parameters used in our model.

Given the scarcity of studies in the Region reporting on average treatment costs for various stages of liver disease, we used cost data from the United States (23) as the basis for calculating the financial savings for healthcare systems. We utilized financial data from Turkmenistan provided by the National Immunization Programme, which estimated annual treatment costs at US$3,500 for acute hepatitis B, US$27,284 for cirrhosis (severe disease), US$13,642 for chronic liver conditions, and US$40,000 for HCC. Although we initially used cost data from the United States as a reference, after consulting with experts, we decided to adopt the treatment cost estimates from Turkmenistan (price year 2024) to calculate the savings for the other countries in our study, as these figures were deemed more regionally appropriate. No additional cost adjustments were applied, as the Turkmenistan estimates were considered the most appropriate available data for the Region.

We developed several scenarios within the model to reflect various programmatic contexts in the countries. The baseline scenario represents the current programmatic aspects, such as vaccination coverage and HBIG treatment, as well as the epidemiological situation in each country. To objectively measure the impact of hepatitis B vaccination on the population, we analyzed three alternative scenarios: a no-vaccination scenario, assuming the absence of an HBV vaccination program, and two optimal scenarios designed to meet hepatitis B elimination targets. These optimal scenarios assume 95% vaccination coverage, 95% screening of pregnant women, and 95% antiviral treatment among those screened.

Optimal Scenario 1 begins in 2021, reflecting the HBV vaccination coverage data available at the time of the study (12, 19, 20). Optimal Scenario 2 starts with the year of introduction of the HBV vaccination program in each of these countries (Belarus: 1996, Georgia: 2001, Kyrgyzstan: 2001, Republic of Moldova: 1995, Turkmenistan: 2002, and Uzbekistan: 2001) to illustrate the potential impact of vaccination. If current vaccine coverage exceeds 95%, we used the most recent data for the optimal vaccination scenario. For Turkmenistan and Uzbekistan, based on the available reports, we assumed 100% vaccine coverage from 2021. In both the optimal scenarios, we also assumed that 95% of newborns from HBV-infected mothers would receive HBIG treatment. The vaccination programs prior to the start of the optimal scenarios mirrored the patterns and coverage rates established in the baseline scenario.

In the baseline scenario, we calculated the prenatal incidence of hepatitis B for each of the birth cohorts based on seroprevalence of hepatitis B in the six countries under study and we have used the same incidence rate as observed in 2024 for all future years. This assumption reflects the absence of significant changes in national screening or antiviral treatment programs in these countries during the projection period, as indicated by recent programmatic reports and expert consultations. To compute the impact using the alternative scenarios in the model, we made the following assumptions: the risk of infection at birth depends on the HBV infection status of pregnant women, their screening status, whether they have received antiviral treatment, and the hepatitis B vaccine coverage and efficacy. When seroprevalence data was not available, we assumed that the HBV prevalence among pregnant women is the same as the general prevalence in the population. In the absence of vaccination 87.5% of infants born to HBV infected women would become infected (16). To align the alternative scenarios with the baseline scenario, we recalculated the prenatal incidence in the baseline by incorporating pregnant women prevalence (the same as the general prevalence in the population), vaccine coverage, and efficacy. Subsequently, we weighted the force of infection for each year to minimize the sum of squared residuals between the incidences obtained from the two methods. These optimal weights were then applied to calibrate the other scenarios.

In all scenarios, if implemented in the country and the birth year, newborns of HBV-positive mothers have the option of undergoing HBIG treatment. We also assumed that the susceptible individuals could get HBV in their lifetime based on the country’s prevalence at each year, their gender and the age of the individuals (21).

We assumed that all vaccinated infants successfully completed the entire dose series. The effectiveness of the vaccination depends on the vaccination coverage (2nd/3rd dose and birth dose coverage) and the efficacy of the vaccine itself. In our presumption, under full vaccination, we considered that all newborns received both the 2nd and 3rd vaccine dose and the birth dose within the first 24 h of birth. Conversely, in the baseline vaccination scenario, we relied on the vaccine coverage data available. The vaccine is assumed to have a 95% efficacy, providing lifelong protection against HBV infection (7, 24–26). In the scenario of having an HBV-infected mother, we assumed the same vaccine efficacy would apply. However, in cases where newborns of infected mothers received HBIG treatment, we presumed a vaccine efficacy of 97% (27).

We assumed that treated individuals would continue to experience liver disease progression, albeit at a reduced rate (28). Evidence from studies of HBV antiviral therapy indicates that treatment lowers the risk of progression to cirrhosis and decompensated liver disease by approximately 40–60% (7, 29–31). On this basis, we assumed a 50% reduction in transition rates in treated individuals. Although fibrosis regression is generally rare and typically occurs only in the early stages of liver disease, most studies indicate that while some degree of regression is possible, it is uncommon, especially in advanced stages (32–34). Therefore, to simplify the model, we chose not to account for fibrosis regression.

### 2.2 Model outcomes

To estimate the full impact of vaccination in preventing severe HBV-related liver disease and death, we simulated birth cohorts spanning from the year of vaccine introduction in each of the studied countries up to 2040 and tracked individuals in each birth cohort throughout their lifetime until death. For instance, if the HBV vaccination was introduced in 2000, we followed the 2000 birth cohort throughout their lives until death. This process was repeated for each subsequent birth cohort from 2001 to 2040. The year 2040 was selected as an arbitrary cut-off to provide a long-term perspective while keeping the analysis scope manageable. For each birth cohort, we calculated the number of individuals who would experience HCC and HBV liver-related deaths under various vaccination scenarios: no vaccination, existing vaccination scenario, and the two optimal scenarios outlined in the previous section. We adapted our progression model for each country based on the factors such as HBV prevalence, vaccination coverage, diagnosis and treatment rates, background mortality, population at birth, and gender distribution. Subsequently, we estimated the percentage of prevented deaths and HCC attributable to each of the vaccination scenarios.

While this analysis offers a comprehensive view of the lifetime impact of HBV vaccination, it does not address the vaccination’s effects on specific calendar years due to the presence of different age groups among infected individuals each year. To address this, we conducted a calendar-based analysis, simulating birth cohorts from 1902 to 2040 and tracking them until death. By combining these cohorts, we derived population figures for each calendar year and calculated liver-related deaths and severe liver disease (including cirrhosis, decompensated cirrhosis, and HCC) for each year based on the vaccination scenarios. For example, in the year 2000, the infected population included both newborns and those born in previous years, with ages ranging from 0 (born in 2000) to 98 (born in 1902).

Simulating cohorts from 1902 ensured comprehensive age distribution coverage at any given time. This method provided a more complete assessment of the vaccination’s impact over time and offered a realistic overview of the current situation in the countries under study.

We also estimated the future burden of hepatitis B and HBV-liver related mortality. In addition, we calculated health care cost savings resulting from sustaining high vaccination coverage as part of the ongoing hepatitis B vaccination program, including treatment costs avoided and the cost associated with time spent in each health state.

## 3 Results

In the first analysis, we estimated the overall impact of vaccination in preventing severe HBV-related liver disease and death by assessing the outcomes of birth cohorts from the year of hepatitis B vaccine introduction up to 2040, tracking these cohorts throughout their lifetime. Key findings from this analysis indicate that existing hepatitis B vaccination in countries can avert a significant percentage of deaths related to hepatitis B in the specified birth cohorts. In Belarus, the hepatitis B vaccination is estimated to prevent around 98.9% of deaths caused by HBV, while in Moldova, the existing hepatitis B vaccination could avert approximately 96.9% of such deaths. Similar estimations in Georgia (98.8%), Kyrgyzstan (98.4%), Turkmenistan (99.1%) and Uzbekistan (97.41%) showcase the effectiveness of the hepatitis B vaccination in reducing HBV-related mortality. Detailed findings, including those related to HCC cases, are presented in Table 2. The estimates indicate the capability of hepatitis B vaccination to prevent 96.3 to 99.2% of HCC cases among individuals born between the year of hepatitis B vaccine introduction and 2040.

**Table 2:** Lifetime impact of HBV vaccination: birth cohort analysis.

Country	Average % (number) of deaths averted^*^			Average % (number) of HCC averted^**^		
	Current vaccination	Optimal scenario 1	Optimal scenario 2	Current vaccination	Optimal scenario 1	Optimal scenario 2
Belarus	98.9% (676)	98.9% (676)	99.53% (682)	98.4% (332)	98.4% (332)	99.35% (335)
Georgia	98.80% (514)	98.81% (514)	98.87% (514)	98.61% (253)	98.62% (253)	98.69% (253)
Kyrgyzstan	98.4% (1346)	98.4% (1346)	99.26% (1356)	98.66% (660)	98.66% (660)	99.36% (664)
Moldova	96.91% (991)	96.92% (991)	98.99% (1017)	96.32% (487.28)	96.33% (487)	98.83% (500)
Turkmenistan	99.1% (1327)	99.1% (1327)	99.4% (1331)	99.22% (656)	99.22% (656)	99.5 (658)
Uzbekistan	97.41% (4786)	97.41% (4786)	99.83% (4862)	98.33% (92503)	98.33% (92507)	99.86% (93964)

* Average death averted across the birth cohorts starting from the year of vaccine introduction in the country and until 2040.

** Average HCC averted in each birth cohorts of starting from the year of vaccine introduction in the country and until 2040.

In the second analysis, we calculated the number of infected population and liver-related deaths for each given calendar year under each vaccination scenario and estimated the future burden of disease and HBV-liver related mortality. In Belarus, without HBV vaccination, our model estimated 2.56% annual infection rates and about 10.60 annual deaths per 100,000 people from 1996 to 2040. The current and optimal vaccination scenarios would prevent 35.33 to 35.77% of infections and reduce deaths to 7.95 to 7.99 per 100,000, averting 24.85 to 25.20% of deaths.

In Georgia, without vaccination, about 2.5% would contract HBV annually from 2001 to 2040, with 14.22 deaths per 100,000. Current vaccination prevents 41.94% of infections, and optimal scenarios prevent 41.94 to 41.98%. Mortality would drop to 10.22 deaths per 100,000, with 26.88 to 26.91% of deaths averted. In Georgia, without vaccination, about 2.5% would contract HBV annually from 2001 to 2040, with 14.22 deaths per 100,000. Current vaccination prevents 41.94% of infections, and optimal scenarios prevent 41.94 to 41.98%. Mortality would drop to 10.22 annual deaths per 100,000, with 26.88 to 26.91% of deaths averted. In Kyrgyzstan, without vaccination, 4.78% of the population would contract HBV annually from 1998 to 2040, resulting in 18.51 annual deaths per 100,000. Current vaccination prevents 38.12% of infections, and optimal scenarios prevent 38.12 to 38.70%. Deaths would decrease to 14.01 to 14.10 per 100,000, with 24.43 to 24.92% of deaths averted. In Moldova, without vaccination, 8.65% would contract HBV annually from 1995 to 2040, with 34.54 annual deaths per 100,000. The current vaccination program prevents 40.17% of infections, and optimal scenarios prevent up to 40.91%. Deaths would fall to 24.02 to 24.59 per 100,000, averting 25.86 to 27.40% of deaths. In Turkmenistan, without vaccination, 5.03% would contract HBV annually from 2002 to 2040, leading to 18.82 annual deaths per 100,000. The current vaccination program prevents 37.71% of infections, and optimal scenarios prevent 37.71 to 37.89%. Deaths would drop to 14.37 to 14.39 per 100,000, with 24.05 to 24.19% of deaths averted. In Uzbekistan, without vaccination, 5.71% would contract HBV annually from 2001 to 2040, with 9.34 annual deaths per 100,000. The current vaccination program prevents 41.95% of infections, and optimal scenarios prevent 41.95 to 43.55%. Deaths would reduce to 7.51 to 7.62 per 100,000, averting 18.47 to 19.66% of deaths. Table 3 summarizes these findings.

**Table 3:** Some key findings (calendar-based analyses).

Country	Average number of deaths per 100,000				Average % of HBV infected cases averted		
	No vaccination	Current vaccination	Optimal scenario 1	Optimal scenario 2	Current vaccination	Optimal scenario 1	Optimal scenario 2
Belarus^*^	10.6	7.9	7.99	7.95^*^	35.323	35.324	35.77
Georgia^**^	14.22	10.22	10.22	10.21	41.942	41.943	41.989
Kyrgyzstan^***^	18.51	14.10	14.10	14.01	38.11	38.12	38.70
Moldova^****^	34.54	24.59	24.59	24.02	40.171	40.176	40.91
Turkmenistan ^*****^	18.82	14.39	14.39	14.37	37.711	37.712	37.89
Uzbekistan ^******^	9.34	7.62	7.61	7.51	41.952	41.954	43.55

* On average and per 100,000 population per year in 1996–2040.

** On average and per 100,000 population per year in 2001–2040.

*** On average and per 100,000 population per year in 1998–2040.

**** On average and per 100,000 population per year in 1995–2040.

***** On average and per 100,000 population per year in 2002–2040.

****** On average and per 100,000 population per year in 2001–2040.

We utilized lifetime cost data from the US (23) and Turkmenistan to calculate the average cost savings from implementing ongoing hepatitis B vaccination programs in comparison to a no-vaccination scenario in each country. In the US-based cost calculations, the cost of treating the acute phase of hepatitis B was not explicitly included. In contrast, for the cost analysis using Turkmenistan’s data, we calculated cost savings both with and without including acute phase treatment costs. As an illustrative example, Table 4 summarizes key findings from the two different data sources for 2016 and beyond, assuming effective treatment for all chronic cases.

**Table 4:** Some key findings (calendar-based analyses).

Country	Cost saved in 2016 (US data)	Average cost saved in 2016–2040 [US data, (23)]	Cost saved in 2016 (Turkmenistan data)	Average cost saved in 2016–2040 (Turkmenistan data)	Cost saved in 2016 (Turkmenistan data)	Average cost saved in 2016–2040 (Turkmenistan data)
Without considering treatment cost in acute phase					With Treatment cost in acute phase	
Belarus	$6.07	$10.71	$14.53	$27.96	$37.349	$37.87
Georgia	$ 3.22	$7.51	8.79	15.16	$23.39	$27.58
Kyrgyzstan	$5.86	$8.30	$15.98	$32.39	$47.718	$68.15
Moldova	$8.22	$15.907	22.30	33.54	$49.57	$57.027
Turkmenistan	$5.47^¶^	$12.03^¶^	$14.95	$32.52	$49.88	$71.68
Uzbekistan	$34.8	$133.6	94.72	216.69	$308.67	$499.168

Treatment cost in million (excluded the treatment for acute phase): Current vaccination vs. no vaccination. Results from the US. cost data.

Findings based on US cost data reveal that in 2016, the ongoing vaccination program in Belarus resulted in cost savings of approximately US$ 6.07 million; In Georgia, there is the potential for an average annual cost savings of US$ 3.22 million. Kyrgyzstan had the potential to save approximately US$ 5.86 million in 2016, while Moldova achieved savings around US$ 8.22 million in the same year. Turkmenistan has saved approximately US$ 5.47 million on medical expenses through its ongoing vaccination program, assuming effective treatment for all chronic cases. Uzbekistan demonstrated a saving of US$ 34.8 million assuming all chronic cases were effectively treated. Over the period from 2016 to 2040, the countries studied have the potential to achieve an average annual cost savings ranging between US$ 7.51 million to $133.6 million, with a mean of $37.4 million and a median of $13.3 million (SD: $49.5 million).

**Figure 2:**
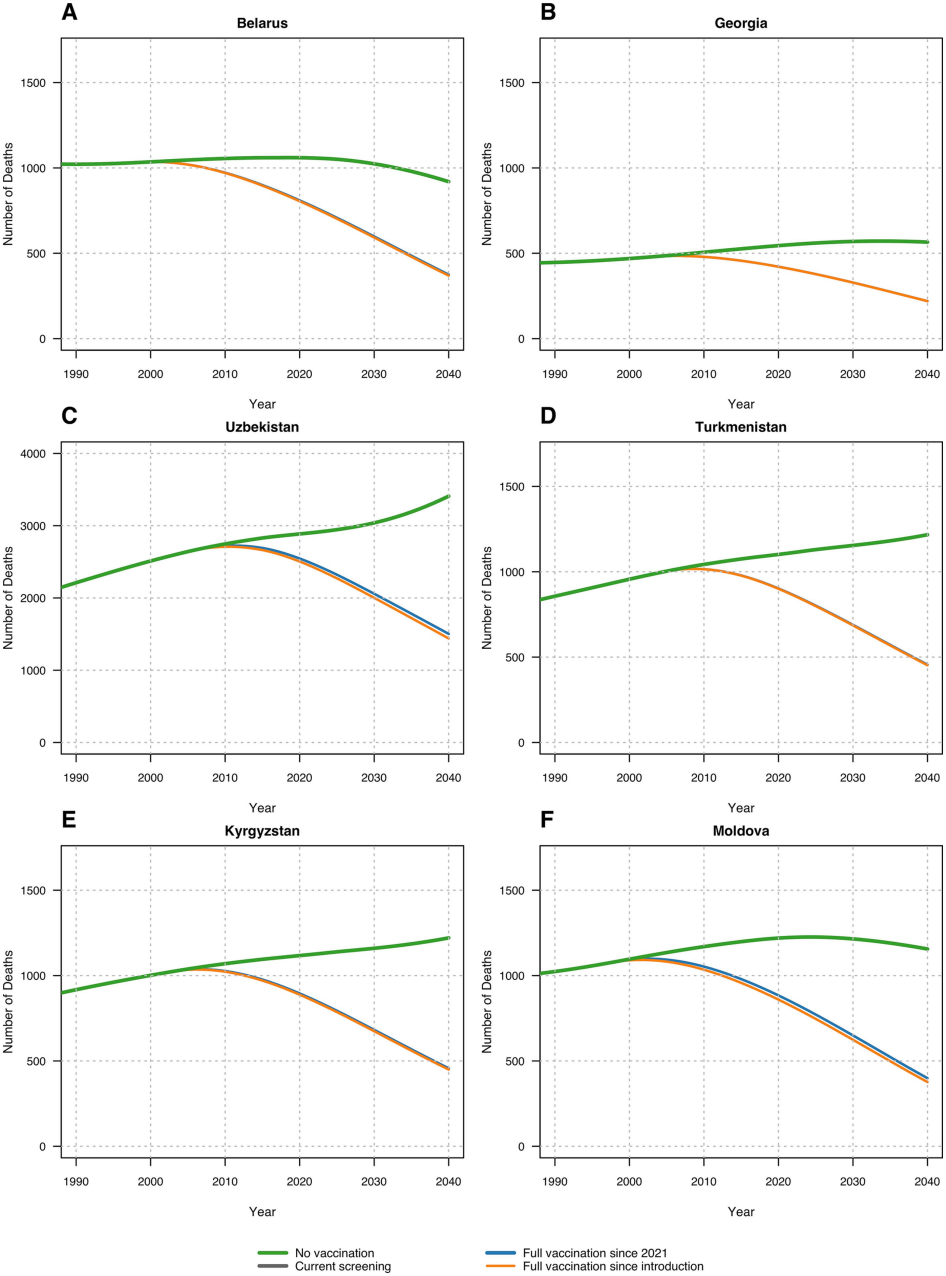
Number of deaths in each scenario across six countries: **(A)** Belarus; **(B)** Georgia; **(C)** Uzbekistan; **(D)** Turkmenistan; (E) Kyrgyzstan; (F) Republic of Moldova.

Figure 2 and Table 4 present the key findings using treatment cost data from Turkmenistan for the years 2016 to 2040 in each country. By utilizing Turkmenistan’s treatment cost data, we also calculated the average annual treatment costs (including acute phase treatment costs) from the introduction of the HBV vaccine in each country up to 2040, estimating the resulting cost savings achieved and projected by the respective Ministries of Health due to reduced treatment needs. In Turkmenistan, without vaccination, the average annual treatment cost was estimated at US$ 99.97 million (95% CI: $97.52 million to $102.41 million). Under the current vaccination scenario, optimal scenario 1, and optimal scenario 2, the country saves approximately US$ 57.20 million, US$ 57.21 million, and US$ 57.41 million annually, respectively. In Georgia, without vaccination, the annual treatment cost would be US$ 42.65 million (95% CI, $42.20 million to $43.11 million). With the current scenario, optimal scenario 1, and optimal scenario 2, the country saves around US$ 23.92 million, US$ 23.92 million, and US$ 23.94 million per year, respectively. In Moldova, without vaccination, the annual treatment cost is estimated at US$ 91.66 million (95% CI, $89.83 million to $93.49 million) for the period from 1995 to 2040. With the current scenario, optimal scenario 1, and optimal scenario 2, Moldova saves approximately US$ 46.40 million, US$ 46.18 million, and US$ 47.48 million annually, respectively. In Belarus, in the absence of vaccination, the annual treatment cost is US$ 73.69 million (95% CI, $70.26 million to $77.12 million) for the years 1996 to 2040. Under the current scenario, optimal scenario 1, and optimal scenario 2, Belarus saves around US$ 32.80 million, US$ 32.80 million, and US$ 33.15 million per year, respectively. In Uzbekistan, without vaccination, the annual treatment cost is estimated at US$ 598.50 million (95% CI, $564.54 million to $632.46 million) for the period from 2001 to 2040. Under the current scenario, optimal scenario 1, and optimal scenario 2, Uzbekistan saves approximately US$ 383.80 million, US$ 383.81 million, and US$ 393.38 million annually, respectively. Finally, in Kyrgyzstan, without vaccination, the annual treatment cost is US$ 94.79 million (95% CI, $92.52 million to $97.08 million) for the years from 2001 to 2040. With the current scenario, optimal scenario 1, and optimal scenario 2, Kyrgyzstan saves around US$ 54.58 million, US$ 54.58 million, and US$ 55.16 million per year, respectively.

**Figure 3:**
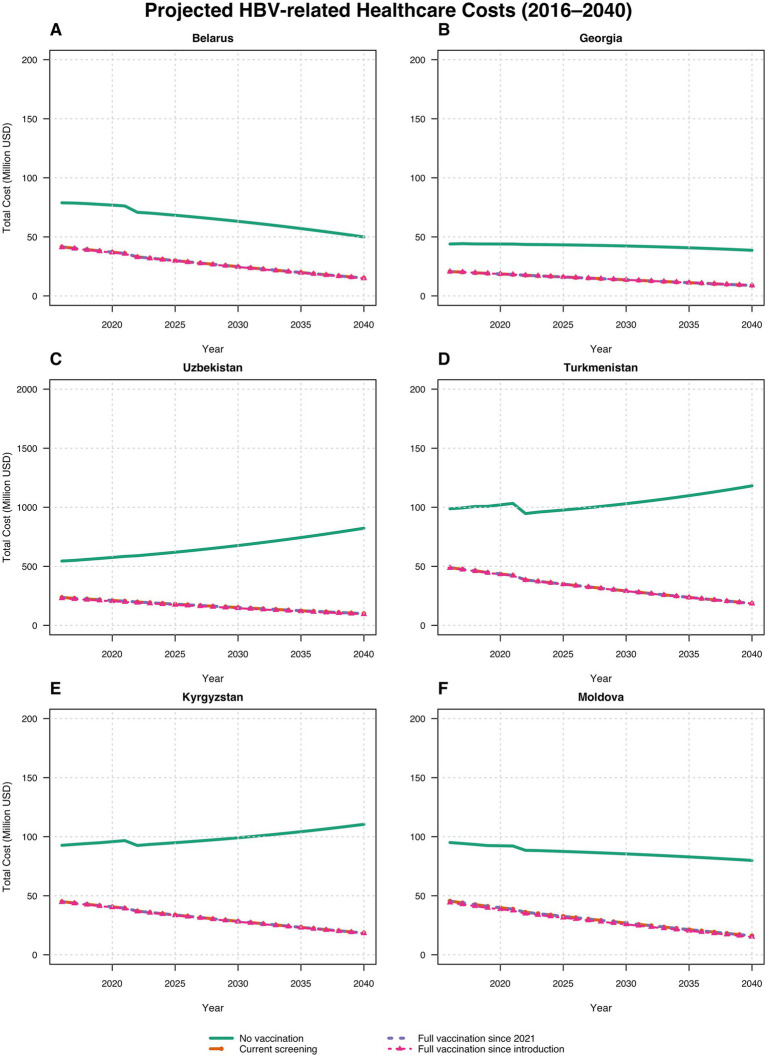
Estimated costs of HBV treatment (including acute phase treatment costs) across scenarios and countries: **(A)** Belarus; **(B)** Georgia; **(C)** Uzbekistan; **(D)** Turkmenistan; **(E)** Kyrgyzstan; **(F)** Moldova. Lines for optimal vaccination scenarios visually overlap because cost differences are small relative to the million USD scale.

These savings underscore the substantial economic benefits from hepatitis B vaccination, highlighting the importance of achieving and maintaining high vaccination coverage thereby reducing healthcare costs and preventing severe HBV-related liver disease (Figure 3).

## 4 Discussion

The progression of an individual from the stage of viral hepatitis to severe liver conditions, such as cirrhosis and HCC, happens over a span of 10 to 20 years (35). Therefore, the impact of hepatitis B vaccination becomes evident after an extended period of time after the introduction of the vaccine in the national immunization schedule. Many research studies have carried out impact analysis of hepatitis B vaccination (7–16). These studies highlight the effectiveness of the hepatitis B vaccination in reducing HBV related morbidity and mortality in various settings. The results of our modeling study are in line with the conclusions from these research studies.

Our birth-cohort analysis, which spans the entire lifespan of individuals, provides a comprehensive assessment of the lifelong impact of hepatitis B vaccination. This analysis offers an overview of the future burden of hepatitis B for each birth cohort. The findings from our modeling study demonstrate that the current hepatitis B vaccination programs in the countries where seroprevalence studies were conducted have led to a remarkable 96.9% reduction in HBV-related deaths over the lifespan of each vaccinated birth cohort. These results offer robust evidence to the Ministries of Health and Finance in the countries of the impact and benefits of investing in immunization programs. Additionally, these findings will empower national immunization program managers to advocate for sustained financing of routine vaccination programs, including making a strong case for the introduction of new and underutilized vaccines into national immunization schedules.

The vaccination scenarios used in our model provide valuable insights into how enhanced hepatitis B prevention programs can significantly reduce the disease burden in countries. According to estimates from the ‘optimal vaccination scenario 2,’ achieving 95% hepatitis B vaccination coverage among infants, combined with screening pregnant women for hepatitis B and treating those who are infected, would prevent a significant number of hepatitis B infections, severe diseases, and deaths compared to a no-vaccination scenario since the introduction of hepatitis B vaccination in national immunization programs.

Estimates from ‘optimal scenario 1,’ which includes additional hepatitis B preventive interventions alongside vaccination, show an impact on hepatitis B disease burden and deaths nearly equivalent to the current vaccination scenario. In 2021, hepatitis B vaccine coverage was already high in the countries, with an average of 92.3% (ranging from 85 to 98%). While the similarity in impact between these scenarios can be attributed to the already high vaccination coverage, the broader benefits of implementing ‘optimal scenario 1’ are likely to become more apparent over a longer period.

These findings provide compelling evidence to incorporate interventions aimed at preventing perinatal transmission of hepatitis B, in addition to existing national prevention programs, thereby leading to substantial healthcare savings.

The calendar-based analysis in our model, spanning from 1902 to 2040, revealed that under the current vaccination scenario, countries can avert 24.08% (95% CI: 21.73–26.43%) of hepatitis B-related deaths from the year of vaccine introduction up to 2040. This finding provides concrete evidence for Ministries of Health on the hepatitis B infections, severe diseases, and deaths prevented each year since the introduction of the hepatitis B vaccine.

Our calendar-based analysis estimates the impact of hepatitis B vaccination from its introduction year only until 2040, which may not capture the entire lifespan of every individual, potentially underestimating the overall impact. It is expected that the long-term effects of current hepatitis B vaccination efforts in reducing diseases and deaths will be significantly higher over an individual’s entire lifetime.

We also calculated the cost savings from the vaccination program using annual lifetime cost data from the United States (23). Additionally, we performed an analysis using treatment costs from Turkmenistan to estimate the treatment cost savings for the countries of our study, given the scarcity of detailed information for each country and the relevance of Turkmenistan’s data compared to the U.S. data. These results may change if annual data becomes available for each country.

Regardless of the cost data used, our analyses underscore the significant economic benefits of hepatitis B vaccination programs across the countries. The results highlight the potential cost savings and the importance of sustained immunization efforts, revealing substantial financial savings achieved and projected by the respective Ministries of Health due to reduced treatment needs under the ongoing vaccination scenario.

The substantial cost savings identified in this study emphasize the importance of maintaining high vaccination coverage. These savings reflect both the treatment costs avoided and the reduced costs associated with time spent in each health state. The data demonstrate that sustained immunization efforts not only improve public health outcomes but also provide significant economic benefits, reinforcing the value of ongoing investment in vaccination programs. These findings could encourage policymakers and healthcare providers to continue supporting and expanding hepatitis B vaccination efforts, particularly in countries with high HBV prevalence. By preventing severe liver disease and reducing the burden on healthcare systems, these programs play an essential role in achieving broader public health goals and ensuring long-term economic sustainability.

### 4.1 Strengths and limitations of the framed model

The architecture of the progression model used in our study is simple yet robust. Our model uses validated and field data from Belarus, Georgia, Kyrgyzstan, the Republic of Moldova, Turkmenistan, and Uzbekistan. Our model was able to estimate the number of deaths prevented due to the hepatitis B vaccination program. In addition, it also assessed the health care cost savings resulting from hepatitis B vaccination. This provided a comprehensive insight of health and economic benefits from hepatitis B vaccination.

Our model has few limitations. The model used in our study is a progression model rather than a transmission model. As a result, we did not account for the indirect effects of hepatitis B vaccination on the population, which could have potentially underestimated the overall impact of hepatitis B vaccination. However, to enhance the accuracy and ensure certainty of our estimates, we used HBV prevalence data for the relevant time period to calibrate the model parameters.

We also simplified the model and did not categorize HBV-infected mothers based on their viral load, due to the unavailability of such data for the included countries. This simplification may have limited the precision of our estimates, as maternal viral load is an important determinant of mother-to-child transmission risk. Future studies could explore this aspect if country-level data become available.

We did not account for the varied rates of protection that may emerge due to variation in hepatitis B vaccination schedules in the countries (36). Due to the high reported immunization coverage by the countries, we assumed that all infants have received their birth dose and subsequent doses of hepatitis B vaccine. However, in reality, there could be instances where the birth dose may have been missed. Considering that high hepatitis B vaccination coverage not only protects the vaccinated individuals but also plays a crucial role in curbing the transmission of hepatitis B within the community, such secondary protection is especially relevant for population groups who are ineligible for vaccination due to various reasons. Our model did not incorporate the impact of vaccine-induced herd immunity, and this also may have resulted in underestimating the overall impact of hepatitis B vaccination. Viral load levels of HBV-infected mothers were not incorporated into the model, as such data were not available for the countries included in the study. This aspect could be explored further in future studies if relevant data becomes available. Moreover, our analysis focused on direct medical costs, excluding non-medical costs and other indirect costs such as lost productivity. Additionally, we did not apply discounting to future costs or benefits, which may have influenced the long-term economic outcomes of hepatitis B vaccination in our model. Also, sensitivity analyses were beyond the scope of this evaluation. However, we recommend this as a direction for future research. Due to the lack of published data on the distribution of HBV viral load among mothers, we simplified the model and did not categorize mothers based on their viral load.

## 5 Conclusion

Despite the limitations of the architecture of the model used in this study, the modeling underscores the substantial impact of hepatitis B vaccination in preventing a considerable number of deaths and severe liver diseases caused by HBV. These results, together with the economic benefits observed, support the view that investing in immunization brings substantial value to public health systems. The longer-term impact of hepatitis B vaccination calls for sustained financing of the national immunization programme to achieve and maintain high immunization coverage for hepatitis B and other vaccines in the national immunization schedule. Such concrete scenario-based estimates devised from vaccine-preventable disease seroprevalence data will support the national immunization technical advisory groups and the Ministries of Health in their decision-making process to introduce new and underutilized vaccines in the national immunization schedule. In addition, this information will also instill confidence in the population of the benefits of vaccines. The health care cost savings from preventing morbidity and mortality from the infectious diseases through vaccination will allow the national health systems to divert financial resources for other acute health needs in the country.

## Data Availability

The original contributions presented in the study are included in the article/supplementary material, further inquiries can be directed to the corresponding author.
